# Thermo-History-Dependent Copper Enrichment During High-Temperature Oxidation of Recycled Steels

**DOI:** 10.3390/ma19030595

**Published:** 2026-02-03

**Authors:** Yuhe Huang, Fangbo Yang, Jun Lu, Shuize Wang, Xinping Mao

**Affiliations:** 1 Institute for Carbon Neutrality, University of Science and Technology Beijing, Beijing 100083, Chinalujun@ustb.edu.cn (J.L.); maoxinping@ustb.edu.cn (X.M.); 2 Institute for Steel Sustainable Technology, Liaoning Academy of Materials, Shenyang 110004, China

**Keywords:** copper enrichment, surface hot shortness, high-temperature oxidation, thermo-history, recycled steels

## Abstract

The utilization of recycled steel is essential for achieving carbon neutrality and sustainable engineering, yet repeated recycling inevitably leads to the accumulation of residual elements that are difficult to remove during conventional refining. Among them, copper (Cu) readily enriches in scrap-based steels and is a primary cause of surface hot shortness during high-temperature processing due to its segregation at the oxide/steel interface. While the compositional effects of Cu have been extensively studied, the influence of thermo-history associated with different industrial processing routes remains poorly understood. In this work, Cu enrichment during high-temperature oxidation was systematically investigated under thermo-histories representative of conventional hot rolling, thin slab continuous casting and rolling (TSCR), and strip casting. Plain carbon steels containing 0.05–0.30 wt.% Cu were oxidized at 1000–1200 °C, and interfacial microstructures were characterized using SEM–EDS. The results show that Cu enrichment is highly sensitive to both temperature and thermal exposure time, with a critical temperature range of 1100–1150 °C promoting the formation of continuous Cu-rich liquid films. Prolonged thermo-history in conventional hot rolling markedly enhances Cu enrichment, TSCR partially suppresses interfacial segregation, whereas strip casting effectively inhibits Cu enrichment even at elevated Cu contents. These findings highlight thermo-history as a dominant factor controlling Cu-induced surface hot shortness and provide guidance for process optimization in recycled steels.

## 1. Introduction

The steel industry is facing increasing pressure to reduce energy consumption and greenhouse gas emissions, prompting a growing interest in the use of recycled steel scrap as a sustainable raw material. Increasing the utilization of recycled steel scrap is widely recognized as an effective pathway to reduce energy consumption and CO_2_ emissions [1]. However, the repeated recycling of steel scrap inevitably leads to the accumulation of residual elements, among which copper (Cu) is the most prevalent and detrimental due to its limited removability during conventional steelmaking processes [2,3]. As a result, Cu-induced surface hot shortness has become a critical bottleneck restricting the quality upgrade and wider application of recycled steels [4,5].

Surface hot shortness in Cu-containing steels originates from selective oxidation during high-temperature processing [6,7,8]. Owing to its lower oxidation potential compared with iron, Cu is rejected from the growing oxide scale and accumulates at the oxide/steel interface. When the local Cu concentration exceeds its solid solubility in austenite, a Cu-rich liquid phase forms within a critical temperature range, typically around 1100–1200 °C [4]. This liquid phase can penetrate along austenite grain boundaries, significantly weakening intergranular cohesion and leading to surface cracking under subsequent mechanical deformation. Such liquid metal embrittlement phenomena have been extensively reported in conventional hot rolling processes. To mitigate Cu-induced hot shortness, various strategies have been proposed, including dilution of scrap, removal of Cu-bearing phases, and alloying additions such as Ni or Si [9,10]. While alloying approaches have shown effectiveness, they are often associated with increased material cost or adverse effects on oxidation behavior and descaling performance. Moreover, most existing studies focus on compositional optimization under simplified thermal conditions, whereas the influence of thermo-history associated with different industrial processing routes remains insufficiently understood.

In industrial practice, steels experience vastly different thermo-histories depending on the manufacturing route. Conventional hot rolling involves long thermal exposure due to reheating and holding at high temperatures, which provides sufficient time for Cu diffusion and interfacial enrichment. Thin slab continuous casting and rolling (TSCR) shortens the thermal cycle by eliminating complete reheating, thereby modifying oxidation kinetics and elemental redistribution [11]. More recently, near-net-shape manufacturing technologies such as strip casting introduce an ultra-short thermo-history, where solidification, rolling, and cooling occur within seconds, potentially suppressing Cu enrichment from a kinetic perspective [12,13]. Despite their increasing industrial relevance, a systematic comparison of Cu enrichment behavior across these representative thermo-histories has not been comprehensively reported.

Unlike most previous studies that focus primarily on the effects of Cu content or alloying strategies to mitigate surface hot shortness, the present work highlights the critical role of thermo-history associated with industrial processing routes. By systematically reproducing representative thermal cycles of conventional hot rolling, thin slab continuous casting and rolling (TSCR), and strip casting, this study demonstrates that Cu enrichment during high-temperature oxidation is governed predominantly by kinetic constraints imposed by thermal exposure history rather than by Cu concentration alone. This process-oriented approach provides new insight into the mechanisms of Cu-induced surface degradation and identifies thermo-history control as an effective pathway for improving surface quality in recycled steels. By reproducing the characteristic thermal cycles of conventional hot rolling, TSCR, and strip casting, the evolution of Cu enrichment at the oxide/steel interface was systematically examined over a broad temperature range, with particular attention to critical temperature windows, Cu concentration thresholds, and the competition between interfacial enrichment and back diffusion into the steel matrix. These findings establish a process-based framework for mitigating Cu-induced surface degradation and underscore the central role of thermo-mechanical processing routes in controlling surface quality in scrap-based steels.

## 2. Materials and Methods

In this study, two experimental material systems were designed to investigate Cu enrichment behavior at the oxide/steel interface. For isolating the effect of Cu content, a series of plain carbon steels with systematically varied Cu concentrations was prepared. The Cu content ranged from 0 to 0.3 wt.%, while the levels of other alloying elements were maintained essentially constant to minimize their influence on Cu enrichment. The steels were produced using a vacuum induction furnace and cast into billets. The chemical compositions of these steels were determined using a full-spectrum spark optical emission spectrometer (ARL iSpark 8860, Thermo Fisher Scientific, Waltham, MA, USA) and summarized in Table 1.

To examine the influence of thermo-history associated with different industrial processing routes, SPA-H steels originating from three representative production lines, conventional hot rolling (CHR), thin slab casting and rolling (TSCR), and strip casting, were employed. Based on their original compositions, 0.3 wt.% Cu was added to each steel. The corresponding chemical compositions are listed in Table 2.

**Figure 1:**
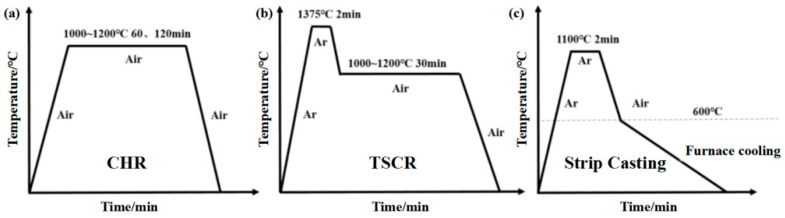
Schematic diagrams of the high-temperature oxidation thermal cycles employed to simulate laboratory reproduction of industrial thermal cycles of different industrial processing routes: (**a**) conventional hot rolling, (**b**) TSCR, and (**c**) strip casting. The distinct thermo-histories highlight differences in peak temperature, holding duration, and cooling behavior.

Plain carbon steels with varying Cu contents were subjected to industrial hot-rolling simulations. Rectangular specimens (~10 mm × 10 mm × 3 mm) were cut from hot-rolled sheets for oxidation experiments. Prior to oxidation, specimens were mechanically ground with SiC papers (400–2000 grit), diamond polished to a mirror finish, ultrasonically cleaned in ethanol, and dried in warm air. The billets were homogenized at 1200 °C for 1 h in a reheating furnace under an argon atmosphere, followed by hot rolling in five passes, reducing the thickness from 40 mm to 3 mm with a finish rolling temperature of 830 °C. After rolling, the sheets were laminar-cooled to 550 °C and then slowly furnace-cooled to room temperature over 24 h. Oxidation weight-gain experiments were conducted to evaluate oxidation behavior and Cu enrichment. For Cu-content-dependent studies, specimens were heated to 1100 °C or 1200 °C under argon and then exposed to laboratory air for 5 min, followed by air cooling. Heating and cooling rates were controlled to reproduce characteristic industrial thermal histories, as schematically illustrated in Figure 1. Oxidation-induced mass gain was measured using an analytical balance. For SPA-H steels, oxidation treatments followed predefined thermal cycles representative of conventional hot rolling, TSCR, and strip casting processes (Figure 1a–c). For microstructural characterization, specimens were sectioned along the rolling direction using wire electrical discharge machining. Longitudinal cross-sections were mechanically prepared by grinding and diamond polishing, followed by 2% nital etching. Microstructures were examined by SEM (TESCAN Mira LMS) at an accelerating voltage of 15 kV and a working distance of ~10 mm. Elemental distributions were analyzed by EDS, and backscattered electron imaging was used to enhance compositional contrast at the oxide/steel interface.

## 3. Results and Discussion

### 3.1. Oxidation Behavior and Copper Enrichment Under Different Temperatures

**Figure 2:**
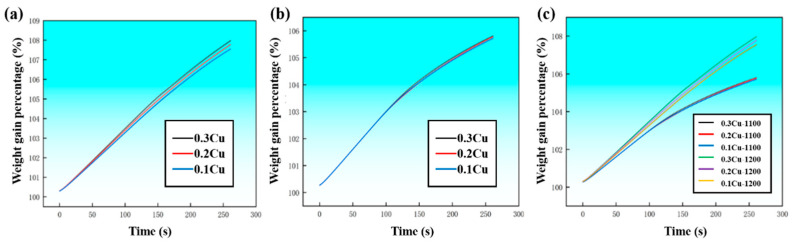
Oxidation weight gain curves of samples containing different Cu contents measured under isothermal oxidation at 1200 °C (**a**) and 1100 °C (**b**), and (**c**) combines the results obtained at both temperatures, enabling a direct comparison of the temperature dependence of oxidation kinetics and the influence of Cu addition on mass gain behavior.

The oxidation behavior of Cu-containing steels was investigated as a function of temperature to understand the fundamental characteristics of Cu enrichment at the oxide/steel interface. Within the examined temperature range of 1000–1200 °C, the oxidation kinetics are predominantly governed by the growth of iron oxides. As shown in Figure 2, all samples exhibit a pronounced increase in oxidation weight gain with increasing temperature, indicating a strong temperature dependence of the oxidation process. Despite variations in Cu content from 0.1 to 0.3 wt.%, no substantial differences in the overall oxidation kinetics or oxide scale thickness are observed at a given temperature, suggesting that residual Cu has a limited influence on the macroscopic oxidation behavior. More specifically, at 1100 °C, the oxidation weight gain curves of steels with different Cu contents nearly overlap, implying that Cu addition does not significantly modify the oxidation rate under this condition. This behavior can be attributed to the fact that oxidation is dominated by Fe diffusion and the formation of iron oxide scales, while Cu, owing to its relatively low affinity for oxygen compared with Fe, does not actively participate in oxide growth [14]. When the temperature is further increased to 1200 °C, the oxidation weight gain increases markedly for all samples. In contrast to the behavior at 1100 °C, a slight but discernible increase in oxidation weight gain is observed with increasing Cu content, although the overall effect remains modest. These results indicate that, within the investigated Cu concentration range, Cu does not substantially alter the oxidation mechanism or kinetics of the steels. Instead, its influence becomes marginally more pronounced only at elevated temperatures, possibly due to enhanced Cu segregation at the oxide/steel interface or localized effects on oxide scale integrity. Nevertheless, the dominant factor controlling oxidation behavior remains temperature, and the oxidation process is primarily governed by iron oxide growth rather than Cu-related reactions.

**Figure 3:**
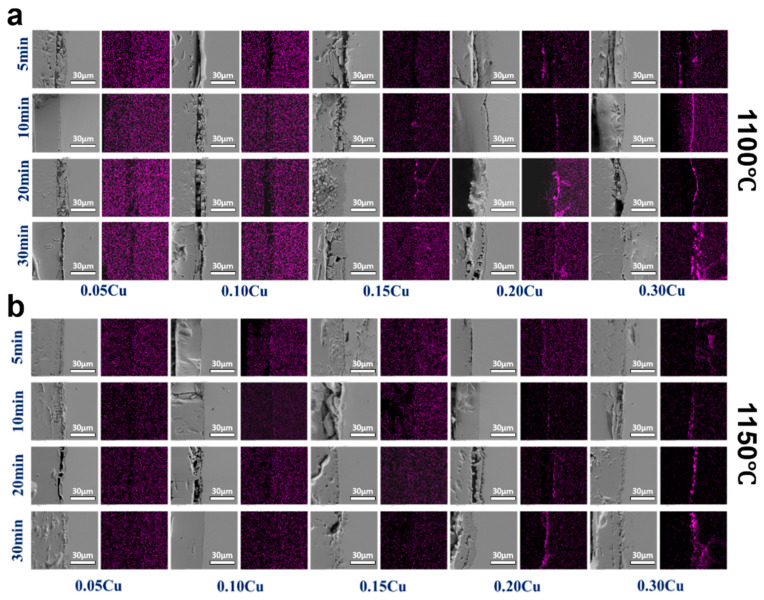
Surface elemental distribution of samples with different Cu contents at (**a**) 1100 °C, and (**b**) 1150 °C.

Although the oxide scale thickness is similar across samples, Cu content appears to have little effect on overall high-temperature weight gain. In contrast, interfacial morphology and elemental distribution, particularly Cu enrichment at the steel/oxide interface, can vary significantly. Literature indicates that 1100–1150 °C is a sensitive temperature range for pronounced Cu segregation, with the extent of enrichment influenced by Cu content, oxidation time, oxidation rate, and reverse diffusion from the surface [4,15]. To quantitatively evaluate the effects of Cu content, temperature, and thermal exposure time on interfacial morphology and Cu redistribution, oxidation experiments were conducted on steels with varying Cu contents at 1100 °C and 1150 °C. The interfacial microstructure and elemental distribution at the oxide/steel interface were examined for steels with different Cu contents after oxidation at 1100 °C for exposure times ranging from 5 to 30 min, as shown in Figure 3a. The Cu enrichment behavior discussed in this study is evaluated based on SEM–EDS elemental mapping performed under identical operating conditions for all samples. While SEM–EDS in Figure 3 provides primarily qualitative to semi-quantitative information, the consistent analytical conditions ensure that comparative trends in Cu enrichment among different thermo-histories can be reliably assessed. At 1100 °C, the steel containing 0.15 wt.% Cu began to exhibit detectable Cu enrichment at the oxide/steel interface after 10 min of oxidation, while clear penetration of Cu along prior-austenite grain boundaries (PAGBs) was observed after 20 min. For steels with higher Cu contents of 0.20 wt.% and 0.30 wt.%, interfacial Cu enrichment was detected throughout the entire oxidation duration (5–30 min). In these samples, both the continuity and intensity of Cu-rich regions increased systematically with increasing oxidation time and bulk Cu content, indicating progressive accumulation of Cu at the interface under prolonged thermal exposure. Figure 3b presents the corresponding interfacial characteristics of samples oxidized at 1150 °C for 5–30 min. In contrast to the behavior at 1100 °C, no obvious Cu enrichment was observed in steels containing 0.05–0.15 wt.% Cu over the investigated time range. Detectable Cu accumulation at the oxide/steel interface emerged only when the Cu content reached 0.20 wt.% or higher, with enrichment already evident after 5 min of oxidation and becoming increasingly pronounced with extended exposure. The observation in Figure 3 demonstrates the existence of a critical Cu content for interfacial enrichment, which is strongly temperature dependent. The threshold Cu level for noticeable enrichment is approximately 0.15 wt.% at 1100 °C, increasing to about 0.20 wt.% at 1150 °C. This upward shift in the critical Cu content suggests that elevated temperature can partially alleviate the enrichment susceptibility of low-Cu steels by enhancing Cu solubility and back diffusion into the steel matrix. However, once the Cu content exceeds this critical level, increasing temperature alone is insufficient to suppress interfacial accumulation, and significant Cu enrichment persists.

### 3.2. Effect of Thermo History Associated with Different Manufacturing Routes

According to the temperature and composition-dependent enrichment behavior discussed above, it is evident that Cu accumulation at the oxide-steel interface cannot be fully rationalized by bulk Cu content and oxidation temperature alone. In practice, steels experience distinct thermo-histories depending on the manufacturing route, which determine not only the peak temperature but also the duration of high-temperature exposure and the continuity of thermal cycles. These factors directly influence oxidation kinetics, interfacial reaction time, and the balance between Cu rejection at the oxide front and its redistribution into the steel matrix. To isolate the effect of thermo-history, Cu enrichment behavior was examined under thermal cycles representative of three typical manufacturing routes, CR, TSCR, and strip casting. Although these routes may operate within comparable temperature ranges, their fundamentally different thermal exposure times provide a basis for assessing how kinetic constraints imposed by thermo-history regulate interfacial Cu accumulation. The following sections analyze the Cu enrichment characteristics associated with each route, beginning with the conventional rolling condition, which represents the longest and most continuous high-temperature exposure.

#### 3.2.1. Effect of Conventional Hot Rolling on Cu Enrichment

**Figure 4:**
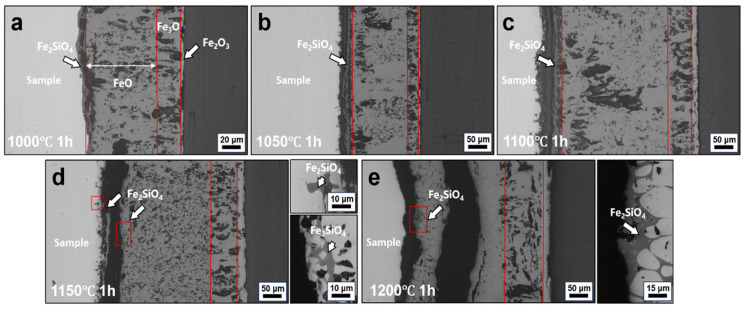
Interfacial morphologies of steels processed by CR after oxidation at different temperatures for 1 h: (**a**) 1000 °C, (**b**) 1050 °C, (**c**) 1100 °C, (**d**) 1150 °C, and (**e**) 1200 °C.

Figure 4 shows the interfacial morphologies of conventionally hot-rolled steels oxidized at 1000–1200 °C for 1 h. Such conditions represent a prolonged thermal exposure typical of industrial slab reheating and hot rolling, where holding times on the order of tens of minutes to several hours are commonly employed, depending on slab thickness and processing schedule. After oxidation, a multilayered oxide scale develops from the oxide/steel interface toward the surface, consisting sequentially of Fe_2_SiO_4_, FeO, Fe_3_O_4_, and Fe_2_O_3_ (for the detailed location, please refer to the position marked by the red line in Figure 4), with the exact phase constitution depending on oxidation temperature and steel chemistry, consistent with previous observations in hot-rolled steel oxidation studies [16]. Under oxidation in air, the long holding time favors the formation of a stratified scale with an inner FeO layer, an intermediate Fe_3_O_4_ layer, and a relatively thin outer Fe_2_O_3_ layer. In some cases, partial decomposition of FeO into FeO + Fe_3_O_4_ is observed during cooling, reflecting the metastable nature of the wüstite layer under non-equilibrium conditions.

As the oxidation temperature increases, pronounced changes in interfacial morphology are observed. At temperatures below 1100 °C, the oxide/steel interface remains relatively planar and compact, and the scale structure is well defined. When the temperature reaches 1150 °C, the Fe_2_SiO_4_ layer disappears, and evidence of liquid phase formation emerges at the interface, accompanied by penetration into both the oxide scale and the steel substrate. This transition indicates a significant change in interfacial stability under prolonged high-temperature exposure and provides favorable conditions for interfacial elemental redistribution. Recent studies on initial oxidation stages of steels at ~1150 °C confirm the formation of liquid phases, which may enhance scale–substrate interaction and promote internal oxidation penetration under industrial thermal cycles [17].

**Figure 5:**
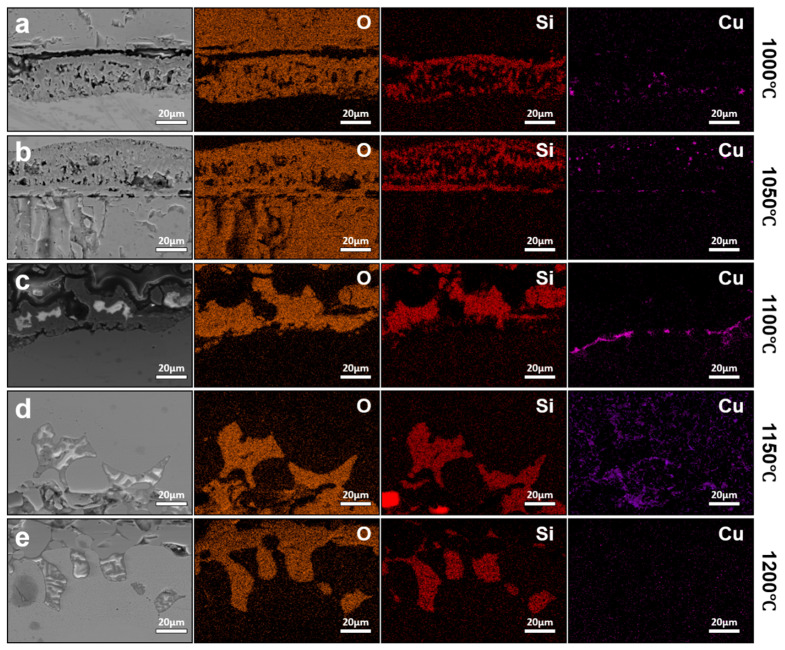
Interfacial elemental distributions of conventionally hot-rolled steels oxidized for 1 h at different temperatures, (**a**) 1000 °C, (**b**) 1050 °C, (**c**) 1100 °C, (**d**) 1150 °C, and (**e**) 1200 °C. The results illustrate the temperature-dependent evolution of Cu enrichment and interfacial phase behavior under prolonged thermal exposure.

The interfacial microstructure and elemental distribution of conventionally hot-rolled steels oxidized at 1000–1200 °C for 1 h were examined, as shown in Figure 5. At oxidation temperatures of 1000 °C and 1050 °C, Cu-rich particles are clearly observed to be retained within the oxide scale rather than accumulating at the oxide/steel interface (Figure 5a,b). At these temperatures, the melting point of Cu-rich phases is not reached, precluding the formation of a liquid phase. Consequently, Cu-rich particles remain mechanically trapped within the growing oxide scale instead of redistributing along the interface. In addition, the presence of Si promotes the formation of internal oxidation products at or near the interface. These internal oxides preferentially form at the junction between Cu-rich particles and the steel matrix, effectively separating Cu-rich phases from the substrate [11]. This process further suppresses interfacial accumulation by anchoring Cu-rich particles within the oxide layer, thereby reducing the amount of Cu available for continuous enrichment at the oxide/steel interface.

When the oxidation temperature is increased to 1100 °C, pronounced Cu enrichment develops along the oxide/steel interface after 1 h of exposure (Figure 5c). Under this condition, the Cu-rich phase becomes liquid and distributes continuously along the interface. Although Fe and Cu share similar crystal structures and comparable metallic atomic radii, which are relevant to their crystallographic compatibility in the austenitic matrix, their markedly different electronic structures significantly limit the solubility of Cu in Fe [18]. The maximum solubility of Cu in austenite is approximately 8.5 wt.%, and this solubility further decreases with increasing carbon content. Once the residual Cu content exceeds the solubility limit in austenite, Cu precipitates preferentially at grain boundaries and exists in a liquid state at elevated temperatures, facilitating interfacial wetting and continuous enrichment. At higher oxidation temperatures, a reduction in interfacial Cu enrichment is observed (Figure 5d,e). After oxidation at 1150 °C for 1 h, the amount of Cu accumulated at the oxide/steel interface is lower than that observed at 1100 °C. At 1200 °C, interfacial Cu enrichment is largely absent. Instead, elemental mapping reveals pronounced penetration of Si into the steel substrate. This behavior can be attributed to the increased solubility and diffusivity of Cu in austenite at higher temperatures, which promote reverse diffusion of Cu from the interface back into the steel matrix, thereby suppressing net interfacial accumulation. Meanwhile, Fe_2_SiO_4_ can react with FeO to form a low-melting eutectic phase, which becomes liquid at approximately 1177 °C. The formation and penetration of this eutectic liquid into the substrate enhances interfacial wetting and oxide adherence [19]. Although this process does not promote Cu enrichment, it can increase the difficulty of subsequent descaling due to stronger oxide–substrate bonding, highlighting a trade-off between suppressing Cu enrichment and maintaining favorable surface processing characteristics at very high temperatures.

**Figure 6:**
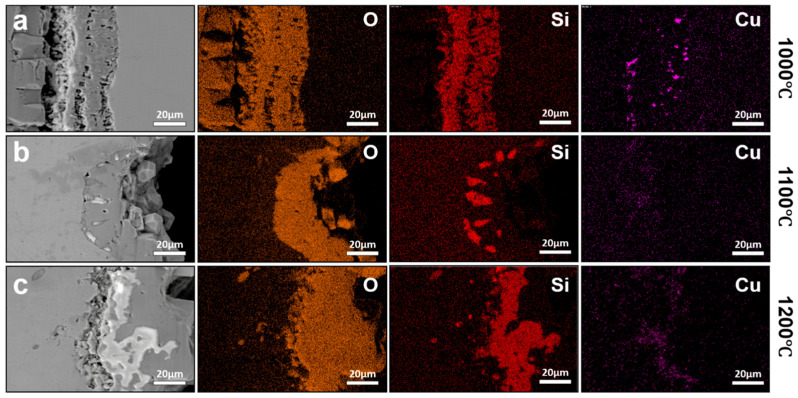
Interfacial elemental distributions of conventionally hot-rolled steels oxidized for 2 h at different temperatures: (**a**) 1000 °C, (**b**) 1100 °C, (**c**) 1200 °C. The results illustrate the temperature-dependent evolution of Cu enrichment and interfacial phase behavior under prolonged thermal exposure.

The effect of prolonged thermal exposure was further evaluated by extending the oxidation time to 2 h over the same temperature range (1000–1200 °C), with the corresponding interfacial features shown in Figure 6. Compared with the 1 h condition, the overall oxide scale morphology remains largely unchanged. At 1000 °C, Cu-rich particles continue to be retained within the oxide layer without forming significant interfacial enrichment. In contrast, at 1100 °C and 1200 °C, the degree of Cu enrichment at the oxide/steel interface is markedly reduced relative to that observed after 1 h.

This attenuation of interfacial enrichment with prolonged exposure can be attributed to time-dependent diffusion effects at high temperature. As oxidation proceeds, the oxidation rate gradually decreases, reducing the rate of Cu rejection from the advancing oxide front. Simultaneously, enhanced bulk diffusion of Cu promotes redistribution from the interface back into the steel matrix. As a result, reverse diffusion progressively dominates over enrichment, effectively suppressing the formation of localized Cu-rich regions at the interface under extended high-temperature exposure.

#### 3.2.2. Effect of TSCR on Cu Enrichment

**Figure 7:**
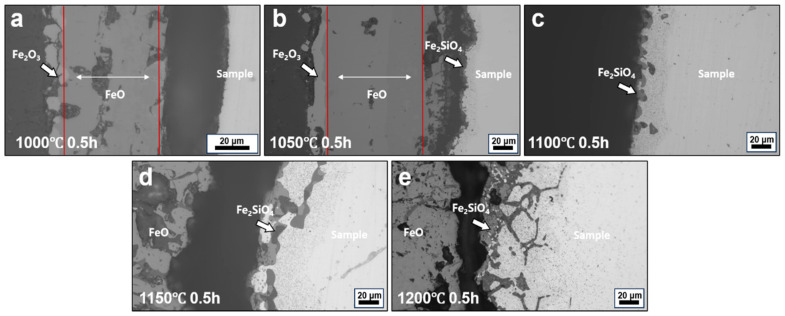
Interfacial morphologies of steels processed by TSCR after oxidation at different temperatures for 0.5 h: (**a**) 1000 °C, (**b**) 1050 °C, (**c**) 1100 °C, (**d**) 1150 °C, and (**e**) 1200 °C.

Compared with conventional hot rolling, TSCR involves a significantly shortened high-temperature exposure by eliminating full slab reheating. This distinct thermo-history is expected to modify oxidation kinetics and limit the time available for interfacial elemental redistribution. Figure 7 shows the interfacial morphologies of steels processed by TSCR after oxidation at 950–1200 °C for 30 min. The lower temperature of 950 °C was selected to represent the lower bound of practical oxidation temperatures encountered during TSCR processing, where steels experience elevated temperatures but with limited holding time. At this temperature, oxidation kinetics are sufficiently active to form a measurable oxide scale, while remaining below the critical temperature range for pronounced Cu enrichment (1100–1150 °C), thereby enabling a clear comparison of Cu enrichment behavior under different thermo-histories. Across the investigated temperature range, the overall macroscopic structure of the oxide scale remains relatively stable. However, when the oxidation temperature exceeds 1100 °C, pronounced penetration of internal oxides into the steel substrate becomes evident. The penetration depth increases progressively with temperature, and at 1200 °C, liquid oxides propagate along PAGBs, forming an interconnected penetration network extending approximately 50–100 μm into the steel matrix.

**Figure 8:**
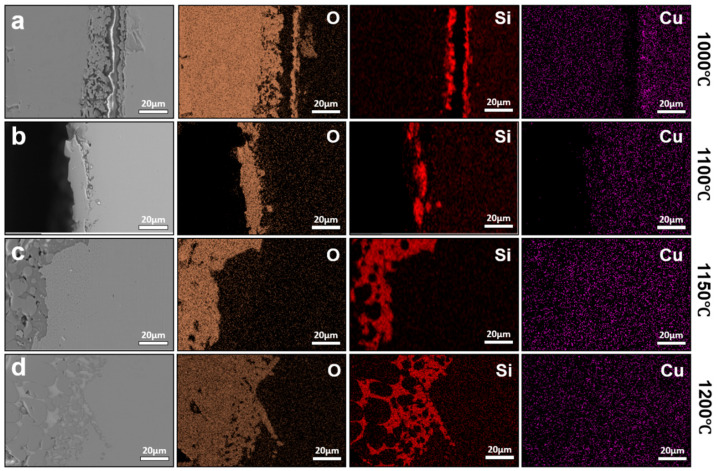
Interfacial elemental distributions of TSCR samples oxidized at different temperatures for 30 min: (**a**) 1000 °C, (**b**) 1100 °C, (**c**) 1150 °C, (**d**) 1200 °C.

Despite these temperature-dependent changes in oxide morphology, the interfacial enrichment behavior of Cu in TSCR samples differs markedly from that observed under conventional hot rolling conditions. Figure 8 presents the interfacial elemental distributions of TSCR samples oxidized at different temperatures for 30 min. In contrast to the conventional hot-rolled steels, no detectable Cu enrichment is observed at the oxide/steel interface over the entire investigated temperature range. This difference can be attributed to two key factors. First, the oxidation duration in TSCR simulations is substantially shorter, limiting the time available for Cu rejection and accumulation at the interface. Second, the TSCR steels contain a lower carbon level, which increases the solubility of Cu in austenite, thereby reducing the tendency for Cu to precipitate and form Cu-rich phases at the interface.

**Figure 9:**
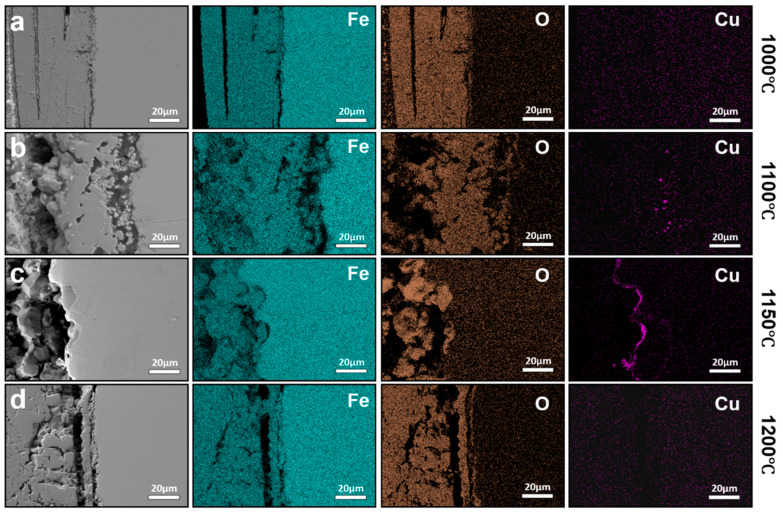
Interfacial elemental distributions of Si-free TSCR samples oxidized at different temperatures, (**a**) 1000 °C, (**b**) 1100 °C, (**c**) 1150 °C, and (**d**) 1200 °C.

Elemental analysis further reveals that the interfacial oxides and penetrated phases are dominated by O and Si, indicating that the internal oxides primarily consist of Fe_2_SiO_4_. With increasing temperature, the Fe_2_SiO_4_ layer becomes progressively thicker. When the temperature remains below 1150 °C, Fe_2_SiO_4_ exists as a solid interfacial layer that partially suppresses further oxidation of the steel substrate and, consequently, limits Cu enrichment. Once the temperature exceeds 1150 °C, Fe_2_SiO_4_ participates in the formation of a liquid phase, which penetrates into the substrate along grain boundaries. This behavior is qualitatively similar to that observed in conventionally hot-rolled steels at comparable temperatures [19,20,21], although the extent of Cu enrichment remains significantly reduced. To further isolate the influence of Si on Cu enrichment behavior, TSCR steels with compositions corresponding to industrial practice but without Si addition were prepared and subjected to identical oxidation treatments. The interfacial elemental distributions of these Si-free TSCR samples are shown in Figure 9. At an oxidation temperature of 1000 °C, no Cu enrichment is observed at the oxide/steel interface. When the temperature increases to 1100 °C, discrete Cu-rich particles appear within the oxide layer, but a continuous Cu-rich interfacial layer, such as that observed in 0.3 wt.% Cu steels oxidized at 1100 °C for 30 min do not form. This difference indicates that, although the effect of Si is eliminated, other residual elements present in the steel, such as Ni (~0.04 wt.%) and P (~0.1 wt.%), also contribute to suppressing Cu enrichment, consistent with previous reports [4]. At 1150 °C, Cu enrichment at the oxide/steel interface becomes clearly detectable in the Si-free TSCR samples, while oxidation at 1200 °C results in a redistribution behavior similar to that observed in conventionally hot-rolled steels, where interfacial Cu enrichment is largely absent. These results demonstrate that, compared with conventional hot rolling, TSCR significantly delays the onset and reduces the severity of Cu enrichment, primarily due to shortened thermo-history and compositional factors that enhance Cu solubility and diffusion back into the steel matrix. Moreover, the comparison between Si-containing (Figure 9) and Si-free (Figure 8) TSCR steels provides further insight into the kinetic factors governing Cu enrichment. In Si-containing steels, the formation of Fe_2_SiO_4_ at the oxide/steel interface plays an important role in modifying oxidation behavior. The Fe_2_SiO_4_ layer acts as a relatively stable interfacial phase, which can partially suppress further oxidation of the steel substrate and reduce the rejection rate of Cu at the advancing oxide front. As a result, the presence of Si effectively increases the kinetic barrier for Cu enrichment under TSCR conditions. In contrast, in the absence of Si, the formation of Fe_2_SiO_4_ is suppressed, leading to a more direct growth of iron oxides and enhanced interfacial reactions. Under these conditions, Cu is more readily rejected from the oxide scale, making interfacial Cu enrichment detectable at elevated temperatures, as observed in the Si-free TSCR samples. Nevertheless, the extent of Cu enrichment remains limited compared with conventional hot rolling, highlighting the dominant role of shortened thermo-history in TSCR. It should also be noted that other residual elements, such as Ni and P, may further influence Cu enrichment behavior. These elements are known to exhibit grain boundary segregation and can compete with Cu for segregation sites, thereby reducing the tendency for Cu accumulation. In addition, Ni is known to stabilize austenite and increase the solubility of Cu in the matrix, which can further suppress interfacial Cu enrichment under high-temperature oxidation conditions.

#### 3.2.3. Effect of Strip Casting on Cu Enrichment

**Figure 10:**
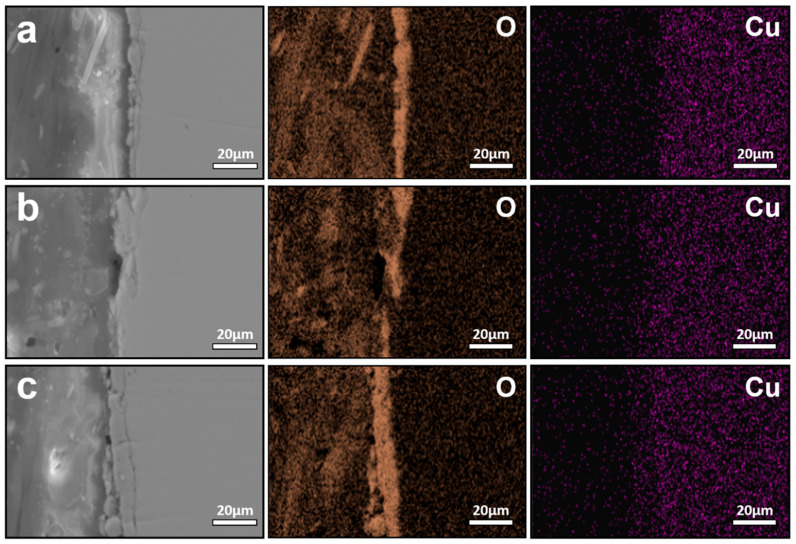
Interfacial elemental distributions of simulated strip-cast samples with different Cu contents, (**a**) 0.1 wt.% Cu, (**b**) 0.2 wt.% Cu, and (**c**) 0.3 wt.% Cu. No significant Cu enrichment is observed at the oxide/steel interface, reflecting the strong kinetic suppression of segregation under ultra-short thermo-history.

Strip casting is a near-net-shape manufacturing route in which molten steel is directly cast and rolled into thin strip within seconds, resulting in an ultra-short thermo-history with virtually no holding at high temperature. Such processing conditions drastically limit oxidation time, diffusion length, and interfacial reaction kinetics. Consequently, strip casting provides an extreme contrast to conventional hot rolling and TSCR, enabling assessment of whether Cu enrichment can be kinetically suppressed even at elevated bulk Cu contents. Figure 10 presents the interfacial elemental distributions of simulated strip-cast samples containing different Cu levels. Regardless of Cu content, no obvious Cu enrichment is detected at the oxide/steel interface. During the simulated strip casting oxidation experiments, the extremely short oxidation duration and relatively lower oxidation temperature result in the formation of a very thin oxide scale, typically less than 5 μm in thickness. Under these conditions, the amount of Cu rejected from the advancing oxide front is insufficient to exceed its solubility limit in the steel matrix. As a result, Cu remains uniformly distributed within the near-surface region rather than segregating at the interface or along grain boundaries. This behavior contrasts sharply with that observed under conventional hot rolling and TSCR conditions, where prolonged or intermediate thermal exposure enables Cu accumulation once critical temperature and compositional thresholds are reached. The absence of interfacial Cu enrichment in strip-cast samples demonstrates that kinetic constraints imposed by ultra-short thermo-history can effectively suppress Cu-induced surface segregation, even in steels containing relatively high Cu levels.

**Figure 11:**
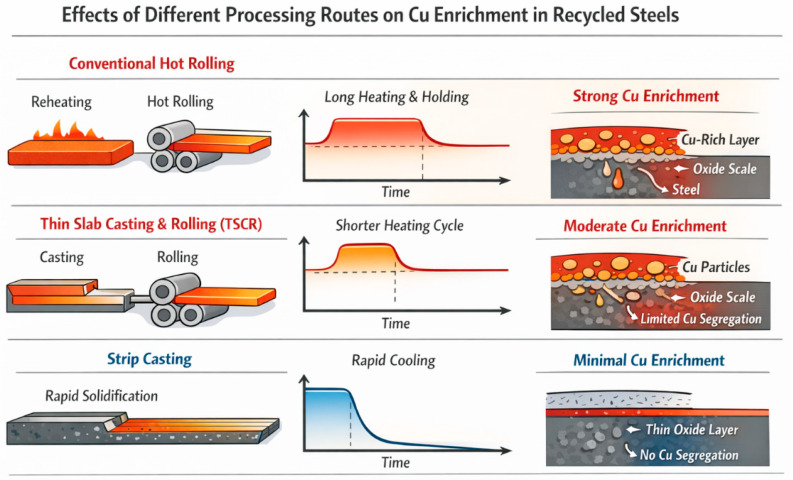
Schematic illustration of thermo-history-dependent Cu enrichment at the oxide/steel interface under different processing routes, showing the formation or suppression of a continuous Cu-rich liquid film during conventional hot rolling, TSCR, and strip casting.

Based on the above experimental observations, the effect of thermo-history on Cu enrichment during high-temperature oxidation can be rationalized from a kinetic–thermodynamic perspective, as schematically illustrated in Figure 11. Although the fundamental driving force for Cu enrichment originates from selective oxidation of Fe and the limited solubility of Cu in austenite, the actual enrichment behavior is dominantly governed by the thermal exposure history associated with different processing routes. In conventional hot rolling, prolonged high-temperature holding provides sufficient time for continuous Cu rejection from the advancing oxide front, enabling the accumulation and coalescence of a Cu-rich liquid film at the oxide/steel interface within the critical temperature range of 1100–1150 °C. In contrast, the shortened thermo-history in TSCR significantly limits the duration of interfacial reactions and Cu diffusion, while enhanced Cu solubility and back diffusion into the steel matrix further suppress continuous interfacial segregation. As a result, Cu enrichment is delayed or manifests only under more severe conditions. Strip casting represents an extreme case of kinetic suppression, where ultra-short thermal exposure and rapid cooling prevent Cu from exceeding its solubility limit near the surface, leading to negligible interfacial enrichment regardless of bulk Cu content. These findings demonstrate that thermo-history plays a dominant role in regulating Cu-induced surface hot shortness, providing a process-oriented pathway to mitigate Cu-related surface degradation in recycled steels without relying solely on compositional modification.

## 4. Conclusions

In this study, the enrichment behavior of residual Cu in recycled steels during high-temperature oxidation was systematically investigated with a focus on thermo-history effects. Cu enrichment at the oxide/steel interface is strongly temperature dependent, reaching a maximum within a critical window of ~1100–1150 °C, where selective oxidation of Fe rejects Cu to the interface and promotes the formation of continuous Cu-rich liquid films once the solubility limit in austenite is exceeded. At higher temperatures, increased Cu solubility and diffusivity enhance back diffusion into the steel matrix, thereby reducing net interfacial enrichment. More importantly, this work demonstrates that Cu enrichment and the associated risk of surface hot shortness are governed predominantly by thermo-history rather than by bulk Cu content alone. Prolonged high-temperature exposure during conventional hot rolling strongly promotes interfacial Cu accumulation, whereas shortened thermal cycles in thin slab continuous casting and rolling significantly delay and suppress Cu enrichment. In contrast, strip casting, characterized by an ultra-short thermo-history and rapid cooling, effectively inhibits interfacial Cu enrichment even at elevated Cu levels. These findings establish thermo-history control as a kinetically effective, process-oriented strategy for mitigating Cu-induced surface degradation in recycled steels and highlight near-net-shape processing routes as a viable pathway for improving surface quality without relying solely on compositional modification. Future work should focus on quantitative modeling of Cu diffusion and enrichment under realistic industrial thermal cycles and on elucidating the synergistic roles of residual elements such as Si, Ni, and P in near-net-shape processing routes.

## Figures and Tables

**Table 1:** Experimental Cu-bearing steel measured chemical composition (wt.%).

No.	C	Mn	Cu	Fe
5Cu	0.067	0.49	0.049	Bal.
10Cu	0.065	0.52	0.097	Bal.
15Cu	0.066	0.51	0.147	Bal.
20Cu	0.063	0.46	0.197	Bal.
30Cu	0.064	0.47	0.289	Bal.

**Table 2:** Measured chemical compositions of the experimental Cu-bearing steels from three different production lines (wt.%).

No.	C	Mn	Si	P	Ni	Cr	Cu	Fe
CR	0.098	0.445	0.437	0.09	0.012	0.403	0.277	Bal.
TSCR	0.066	0.494	0.477	0.09	0.044	0.557	0.298	Bal.
Castrip	0.046	0.778	0.213	0.09	0.025	0.437	0.222	Bal.

## Data Availability

The original contributions presented in this study are included in the article. Further inquiries can be directed to the corresponding authors.
